# Retraction Note: Diagnostic and prognostic value of plasma microRNA-195 in patients with non-small cell lung cancer

**DOI:** 10.1186/s12957-019-1664-y

**Published:** 2019-07-10

**Authors:** Keli Su, Tingcui Zhang, Yongrui Wang, Guijun Hao

**Affiliations:** 1 Department of Oncology, The Fourth People’s Hospital of Jinan, NO. 50, Shifan Road, Jinan, 250031 Shandong Province China; 2 Department of Internal Medicine, The Central Hospital of Jinan, Jinan, 250012 Shandong Province China; 3 Department of Clinical Laboratory, The Fourth People’s Hospital of Jinan, Jinan, 250031 Shandong Province China

**Retraction Note: World J Surg Oncol**

**https://doi.org/10.1186/s12957-016-0980-8**

This article [1] is retracted at the request of Editor-in-Chief due to overlap with articles [2–5].

None of the authors have responded to any correspondence from the editor or publisher about this retraction.
